# Kidney mRNA-protein expression correlation: what can we learn from the Human Protein Atlas?

**DOI:** 10.1007/s40620-024-02126-z

**Published:** 2024-11-11

**Authors:** Dianne Acoba, Anna Reznichenko

**Affiliations:** 1 Clinical Renal, Late-Stage Development, Cardiovascular, Renal and Metabolism (CVRM), BioPharmaceuticals R&D, AstraZeneca, Gothenburg, Sweden; 2 Institut Necker Enfants-Malades (INEM), Institut National de La Santé et de La Recherche Médicale (INSERM) U1151, Université Paris Cité, Paris, France

**Keywords:** Human Protein Atlas, Immunohistochemistry, RNA-seq, Kidney

## Abstract

**Background:**

The Human Protein Atlas, with more than 10 million immunohistochemical images showing tissue- and cell-specific protein expression levels and subcellular localization information, is widely used in kidney research. The Human Protein Atlas contains comprehensive data on multi-tissue transcript and protein abundance, allowing for comparisons across tissues. However, while visual and intuitive to interpret, immunohistochemistry is limited by its semi-quantitative nature. This can lead to mismatches in protein expression measurements across different platforms.

**Methods:**

We performed a comparison of the Human Protein Atlas’ kidney-specific RNA sequencing and immunohistochemistry data to determine whether the mRNA and protein abundance levels are concordant.

**Results:**

Our study shows that there is a discordance between mRNA and protein expression in the kidney based on the Human Protein Atlas data. Using an external validation mass spectrometry dataset, we show that more than 500 proteins undetected by immunohistochemistry are robustly measured by mass spectrometry. The Human Protein Atlas transcriptome data, on the other hand, exhibit similar transcript detection levels as other kidney RNA-seq datasets.

**Conclusions:**

Discordance in mRNA-protein expression could be due to both biological and technical reasons, such as transcriptional dynamics, translation rates, protein half-lives, and measurement errors. This is further complicated by the heterogeneity of the kidney tissue itself, which can increase the discordance if the cell populations or tissue compartment samples do not match. As such, shedding light on the mRNA-protein relationship of the kidney-specific Human Protein Atlas data can provide context to our scientific inferences on renal gene and protein quantification.

**Graphical abstract:**

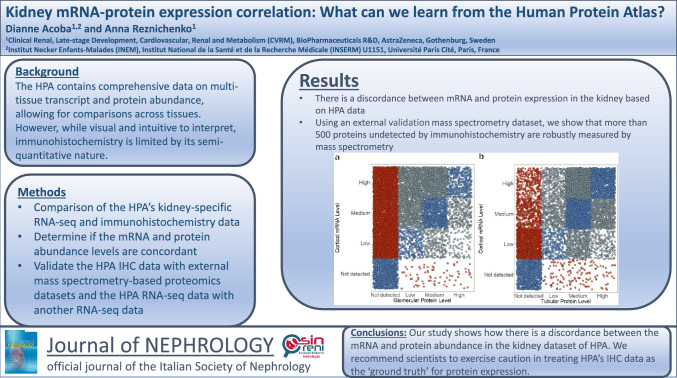

**Supplementary Information:**

The online version contains supplementary material available at 10.1007/s40620-024-02126-z.

## Introduction

The complexity of chronic kidney disease (CKD) necessitates a non-reductionist systems biology approach to understand its multifactorial biology, making high-throughput or omics studies an important pillar in kidney research [1]. Reference datasets are essential for benchmarking healthy expression levels and the Human Protein Atlas is invaluable as it provides both transcript and protein abundance information.

The Human Protein Atlas is widely used in kidney research, with more than 2000 CKD research articles citing the Human Protein Atlas’ seminal publication in 2015 [2]. Common uses of the Human Protein Atlas include checking tissue- and cell-specific mRNA and protein expression levels, subcellular localization, and protein structure and interactions. Other existing resources also provide kidney transcript and protein expression data, however, the Human Protein Atlas is currently the only platform that contains multi-tissue transcript and protein abundance information, which uniquely allows for a head-to-head comparison between mRNA and protein levels. It is also continuously being updated with new information as technologies emerge.

The Human Protein Atlas’ renal transcriptome data are derived from deep sequencing of mRNA (RNA-seq) of frozen kidneys of nine individuals, six females (48–67 years old) and three males (46–78 years of age). The kidney samples are composed of heterogeneous cell populations, accounting for 60–80% tubules, 5–25% glomeruli, 5–10% fibroblasts, and 5–25% other cell types [2]. RNA-seq allows for global and unbiased transcript identification and quantification in a single high-throughput sequencing assay, compared to the limited and targeted nature of microarray technologies. With RNA-seq, isoforms and differential exon usage can be measured, sequencing depth and precision are better compared to earlier technologies, and absolute quantification is possible [3]. On the other hand, the kidney protein expression dataset is derivedfrom antibody-based protein profiling through conventional brightfield immunohistochemistry (IHC) for normal kidney tissue. Tissue microarrays are stained with DAB (3,3’-diaminobenzidine)-labeled antibodies and counterstained using hematoxylin. The kidney proteome is represented by samples from three individuals (not uniform across all proteins) and IHC provides semi-quantitative and relative measurements that include information on the staining intensity, quantity, and location.

Previous studies exploring mRNA-protein expression relationships in different human tissues, including the kidney, showed a surprising lack of strong correlations [4–6]. Variability in transcript-protein correlation has been observed across tissues (within-gene correlation), and even across genes (across-gene correlation). The variability is postulated to be due to spatial and temporal mRNA dynamics, post-transcriptional and post-translational regulation, translation rates, protein synthesis constraints and delay, protein transport and half-lives, and potential measurement error and technical variability [3, 7]. The kidney is not exempt from this correlation discordance, primarily driven by the tissue’s complexity and highly heterogeneous cell populations [8]. Tissue complexity leads to incorrect estimates of mRNA-protein correlations if the fraction of different cell types in the samples are unmatched [3].

In this study, we compare Human Protein Atlas kidney mRNA and protein expression data, which has not been done previously, utilizing a semi-quantitative proteomics dataset. Analyzing the kidney mRNA-protein relationship using Human Protein Atlas data will allow us to infer better biological insights and to interpret our research results more accurately.

## Methods

Kidney protein and transcript expression data were downloaded from the Human Protein Atlas v23.0 on February 1, 2024 [2]. The protein expression data provided antibody reliability (“Approved”, “Enhanced”, “Supported”, and “Uncertain”), semi-quantitative expression level (“High”, “Medium”, “Low”, and “Not detected”), and cell type (“bowman’s capsule”, “cells in glomeruli”, “cells in tubules”, “collecting ducts”, “distal tubules”, “proximal tubules (cell body)”, and “proximal tubules (microvilli)”) information for 13,467 proteins. Protein data were then filtered to exclude entries with “Uncertain” antibody reliability, and to only include genes with glomerular (“cells in glomeruli”) and tubular (“cells in tubules”) expression data, as the other cell types have limited protein measurements.

To classify transcript expression data from RNA-seq according to levels similar to the protein expression dataset, nTPM (normalized transcripts-per-million) values equal to zero were marked as “Not detected” and the remaining were divided into tertiles and consequently classified as low, medium, or high. Transcript and protein data were plotted for comparison; genes and proteins with concordant and discordant mRNA-protein levels were identified.

As external reference datasets, kidney compartment-specific mass-spectrometry proteomics data from the Kidney Precision Medicine Project (KPMP) PXD033207 [9] and healthy control RNA-seq transcriptomics data from Levin et al. (2020) [10] were used. All analyses were performed in R 4.3.1 [11].

## Results

In the transcript expression data, 20,162 transcripts are measured in the kidney. Of these, 3764 are considered not detected (normalized transcripts-per-million = 0) and the high, medium and low expression tertiles have 5481, 5452, and 5465 transcripts, respectively. High expression is defined as 13.3–52,063.1 normalized transcripts-per-million, medium expression as 3.0–13.3 normalized transcripts-per-million, and low expression as 0.1–3.0 normalized transcripts-per-million. A total of 11,157 mRNA-protein pairs in the glomeruli and 10,108 in the tubules are used in subsequent analyses. In the glomerular IHC data, 5988 proteins are not detected, and 2078, 2240 and 851 proteins have low, medium, and high expression, respectively. For the tubular IHC data, 3080 proteins have no expression detected, while 1201, 3666 and 2161 proteins have low, medium, and high expression, respectively.

**Fig. 1:**
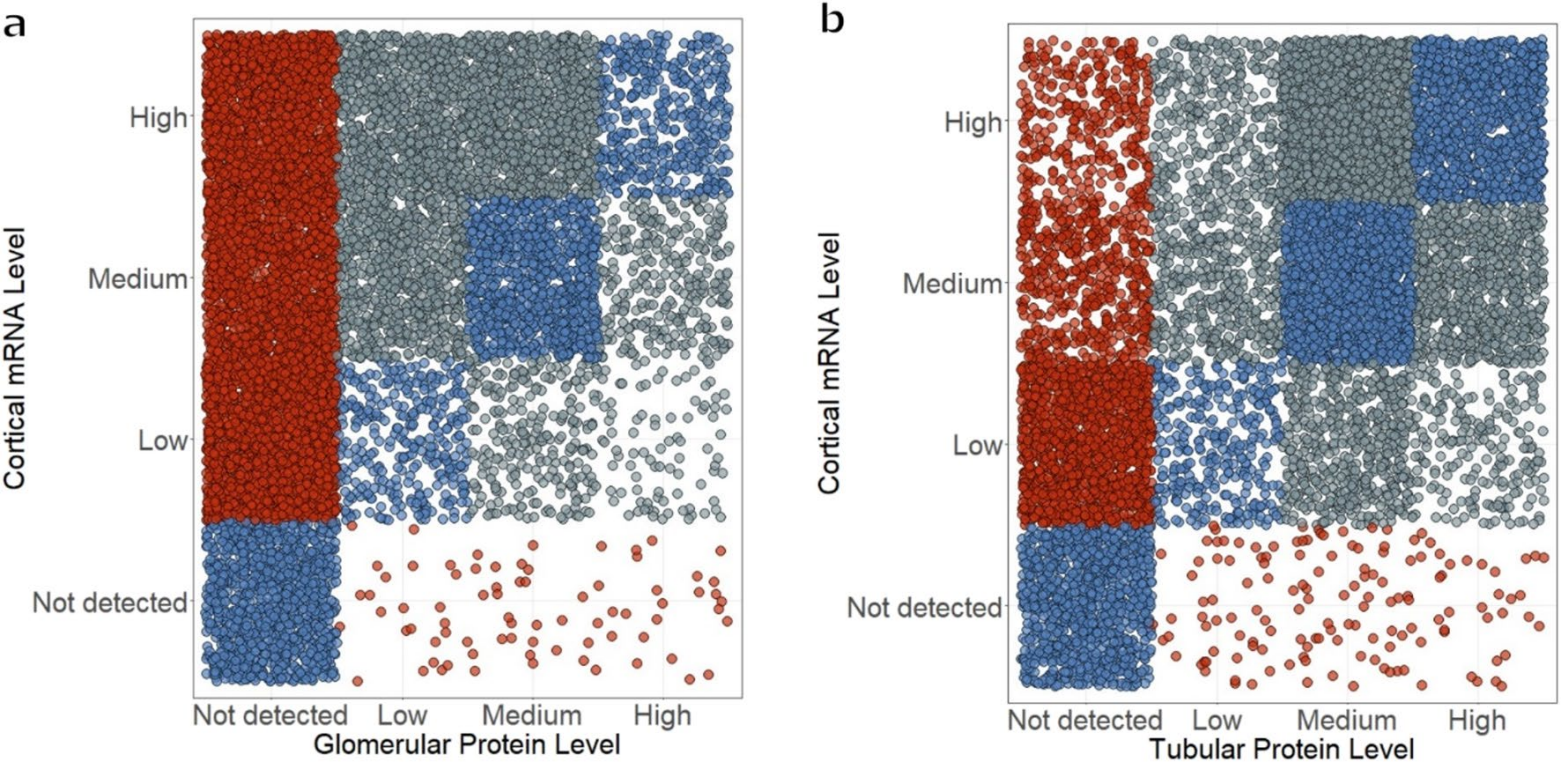
Kidney tissue mRNA & protein abundances. Stripcharts demonstrate the correspondence between the mRNA and protein semi-quantitative expression levels in the **a** glomeruli and **b** tubules. Each individual dot represents one gene with both RNA and protein expression quantified. Perfectly concordant mRNA-protein pairs are shown with blue fill, the extremely discordant pairs are in red, and gray fill reflects the in-between scenarios

To visualize how the transcript and protein expression reported in the Human Protein Atlas correlate, kidney mRNA expression data were plotted against both glomerular and tubular IHC data (Fig. 1a-b). Approximately 24% (2682 pairs) of the glomerular and 38% (3808 pairs) of the tubular mRNA-protein pairs show expression abundance agreement, while there are 8472 pairs (75.93%) with discordant protein-mRNA abundance in the glomeruli and 6298 (62.31%) in the tubules (Table 1). Despite this, statistical testing via chi square test shows that mRNA and IHC data are not independent of each other, indicating that the two are associated. Of particular interest to us are those with undetected protein levels but measurable mRNA expression and vice versa, those with unmeasured mRNA but detectable protein levels. In both the glomeruli and tubules, there are more undetected proteins with observable mRNA expression. In the glomeruli, 4953 proteins (44.39%) are undetected but have measurable mRNA and the same holds for 2122 proteins in the tubules (20.99%), while with regard to proteins with IHC signals but no detectable mRNA expression, 76 (0.68%) are in the glomeruli and 141 (1.39%) are in the tubules.

**Table 1:** Count summary of the kidney proteins with discordant mRNA-protein abundance levels in the Human Protein Atlas

	Glomeruli	Tubules
Number of reliable paired mRNA-protein expression measurements	11,157	10,108
mRNA-protein pairs with concordant abundance levels	2682 (24.04%)	3808 (37.67%)
mRNA-protein pairs with discordant abundance levels	8475 (75.96%)	6300 (62.33%)
mRNA-protein pairs with no IHC signals but with detected kidney mRNA expression (nTPM > 0)	4953 (44.39%)	2122 (20.99%)
mRNA-protein pairs with no IHC signals but with detected kidney mRNA expression (nTPM ≥ 1)	3773 (33.46%)	1172 (11.59%)
mRNA-protein pairs with no IHC signals but with detected kidney mRNA expression (nTPM > 0) and measured using compartment-specific mass-spectrometry data (mean spectral count ≥ 1)	637 (5.71%)	171 (1.69%)
mRNA-protein pairs with no detectable mRNA but with IHC signals	76 (0.68%)	141 (1.39%)
mRNA-protein pairs with no detectable mRNA but with IHC signals and detected by compartment-specific RNA-seq (TPM > 0)	62 (0.56%)	120 (1.19%)
mRNA-protein pairs with no detectable mRNA but with IHC signals and detected by compartment-specific RNA-seq (TPM ≥ 1)	5 (0.04%)	3 (0.03%)

**Fig. 2:**
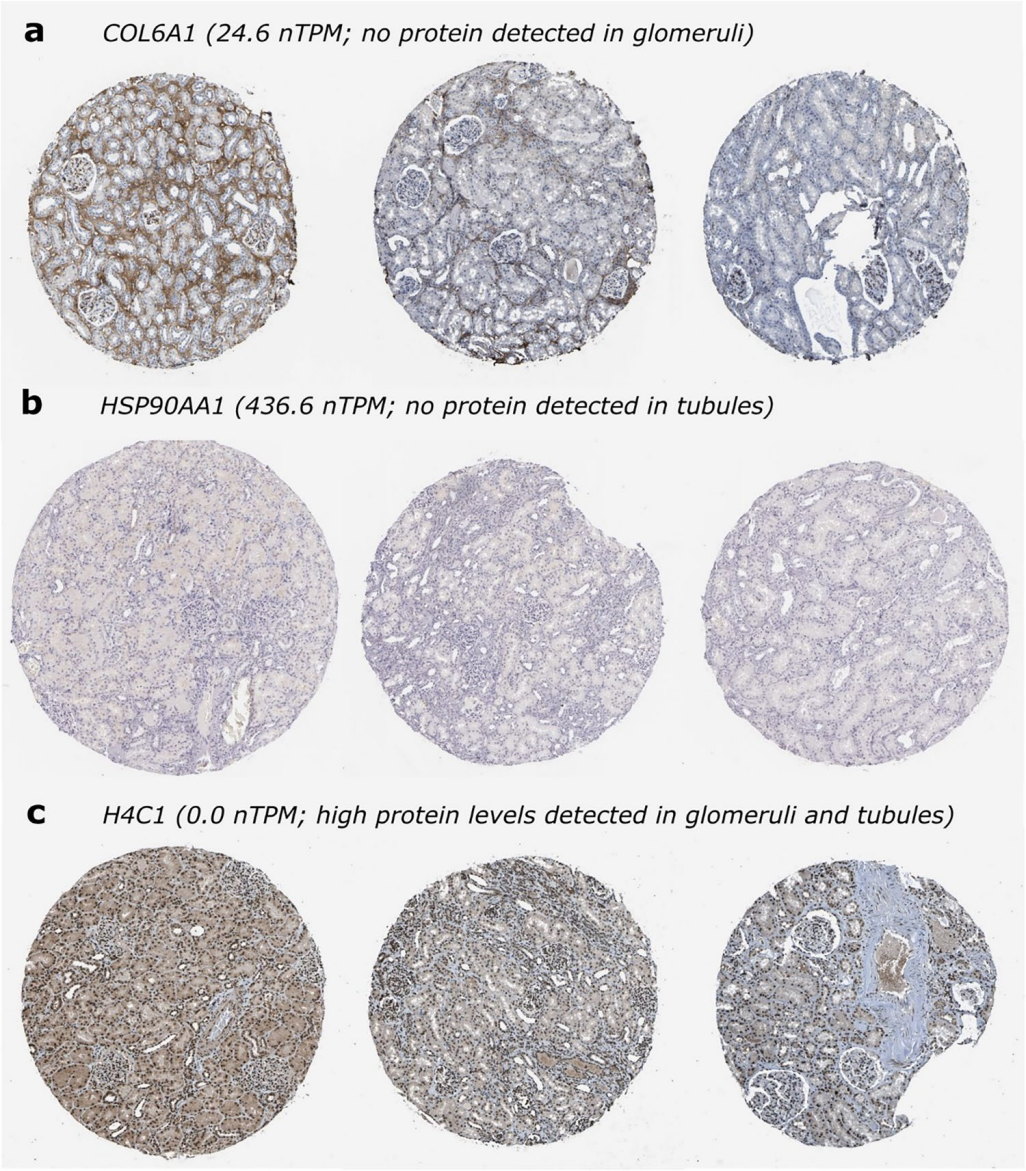
Representative immunohistochemical images of proteins with extremely discordant mRNA-protein expression levels according to the Human Protein Atlas. **a**, **b** Immunohistochemical images showing high glomerular COL6A1 (**a**) and tubular HSP90AA1 (**b**) protein expression. **c** Immunohistochemical images showing no detectable glomerular and tubular protein expression for H4C1*.* Image credit from the Human Protein Atlas. Images are available from https://v23.proteinatlas.org

Examples of extremely discordant pairs with respect to mRNA and protein expression are shown in Fig. 2. *COL6A1*, which encodes for collagen type VI alpha 1 chain and is involved in extracellular matrix organization, is not detected in the glomeruli at the protein level through immunohistochemistry (Fig. 2a) but has high transcript expression in the kidney (24.6 normalized transcripts-per-million) and specifically, the glomeruli (18 transcripts-per-million), according to the Human Protein Atlas and Levin et al. (2020), respectively. The same mRNA-protein abundance measure is observed for *HSP90AA1* encoding for the molecular chaperone heat shock protein 90 alpha family class A member 1 (Fig. 2b) in the tubules (436.6 normalized transcripts-per-million in the Human Protein Atlas, 202 transcripts-per-million in Levin et al.). On the other hand, *H4C1*, encoding for H4 clustered histone 1, exhibits high protein expression as measured by IHC in both glomeruli and tubules (Fig. 2c) but has very low to almost no detectable mRNA expression in both the Human Protein Atlas and Levin et al. (2020) RNA-seq data.

As a cross-platform validation analysis, we tested whether the proteins with no IHC signals despite having mRNA expressed can be detected by mass spectrometry. Using healthy glomerular and tubulointerstitial proteomics data, we determined that 637 of these IHC-undetected glomerular proteins and 171 tubulointerstitial proteins are detected by mass spectrometry with mean spectral count ≥ 1 (Table 1; Online Resource 1). COL6A1 and HSP90AA1, despite having no discernible immunohistochemical staining (Fig. 2a–b), have robust protein expression in the glomeruli (82 mean spectral count) and tubulointerstitium (97 mean spectral count), respectively, according to the KPMP mass spectrometry data.

Of the 76 glomerular and 141 tubular proteins with no detectable transcript expression, 62 and 120 have measurable mRNA expression based on Levin et al. (2020) RNA-seq data, respectively. However, transcript expression is generally low, only 5 glomerular and 3 tubulointerstitial transcripts have mean transcripts-per-million ≥ 1 (Table 1; Online Resource 1).

## Discussion

Immunohistochemistry allows for intuitive visual identification and localization of a target protein at both cellular and subcellular levels. It is routinely used in clinical practice for diagnostic pathology and is an important tool not just in healthcare but also in research. The Human Protein Atlas project has performed high-throughput IHC to map the human proteome in tissues and cells [2]. However, the semi-quantitative nature and narrow range of staining intensity of IHC pose limitations in its usage. We hence investigated if there is a disparity in the immunohistochemical and RNA-seq datasets in the Human Protein Atlas kidney-specific proteome and to compare IHC-reported protein expression levels with kidney proteomics data.

We observed better concordance between the kidney mRNA and tubular IHC data than its glomerular counterpart, which could be due to the RNA-seq samples being 60–80% tubules. Kidney biopsies are mostly composed of cortical tissues, which primarily contain tubular cell populations. Cross-checking the Human Protein Atlas’ IHC data with external mass spectrometry data, we found that some undetected proteins by IHC are expressed and detected in other datasets. On the other hand, the Human Protein Atlas’ RNA-seq data seem to be more accurate as most of the undetected transcripts also have very low transcript expression in a validation dataset. This is expected as the Human Protein Atlas RNA-seq data are mostly consistent with other human transcriptome datasets, such as GTEx and FANTOM5 [12].

Of interest are the mRNA-protein pairs with extremely discordant expression. In a first scenario, where no mRNA is detected but protein expression is high, the discordance could be due to non-specific staining brought about by a promiscuous polyclonal antibody or IHC protocol failure, especially in the blocking step. The antibody could have low binding affinities leading to dissociation during processing steps [13]. The epitope could also be located in a cellular compartment inaccessible to reagents, or tissue artifacts could be present leading to false-positive staining due to leakage of proteins [13]. In the tubular cells of the kidney, proteins are reabsorbed and as such, could bind to antibodies non-specifically. For example, *H4C1* is highly expressed at the protein level according to IHC despite practically being undetected transcript-wise (Fig. 2c) and the Human Protein Atlas explains that this could be due to the antibody targeting proteins from more than one gene. The other scenario is when transcript expression is detected but immunohistochemistry does not detect any protein expression. This could be due to a weak or diluted antibody and a failure in the antigen retrieval protocol [13]. It could also be due to a difference in the expected location of the transcript and the protein, which is what is inferred about *COL6A1* (Fig. 2a). These are just examples of how both biological and technical reasons could bring about such differences in abundance levels of the transcript and protein.

In this study, we only focused on the glomerular and tubular data and excluded the others, which are a minority. However, this makes mRNA-IHC comparison even trickier due to potential variability in patient tissue samples and sample preparation differences. This is a limitation of both this study and of the Human Protein Atlas: the samples used for IHC and RNA-seq are different, thus preventing us from comparing the same biosamples. Despite being considered histologically normal, the tissue samples are not from healthy individuals, who may have underlying diseases that could affect kidney molecular processes and protein and transcript expression. Additionally, the Human Protein Atlas has a disclaimer on their website about how an antibody may not bind to its target due to differences in protein conformation and target accessibility. Protein denaturation and concentration, as well as sample complexity, are some of the factors that may influence off-target binding, which could lead to false results [2]. The Human Protein Atlas solves this issue by providing a reliability score to its data, and also the reason why we chose to exclude all proteins with “uncertain” reliability in this analysis.

Despite the depth of characterization housed by the Human Protein Atlas, immunohistochemistry comes with limitations. Aside from its reliability being dependent on the antibodies used, it is also only a semi-quantitative measurement, manually interpreted by a pathologist, which can cause interobserver variability. Reproducibility also hinges on laboratory protocols. The most substantial hurdle is the narrow linear range of its staining intensity, resulting in rapid assay saturation [14]. The single order of magnitude range of the antibody label DAB, for example, is poorly suited to assess markers across the full dynamic range of biological protein expression, which is approximately 8 orders of magnitude [4].

Immunohistochemistry provides spatio-temporal expression information at a cellular or subcellular level, although at the price of quantitative measurements [15]. Mass spectrometry methods can measure the dynamic protein expression range and is an important complement to IHC, by also providing isotype-specific information [4, 16]. However, mass spectrometry has low sensitivity and is biased toward more precise detection of abundant proteins [16]. The current development and improvement of high-throughput single-cell spatial proteomics methods, however, is one of the potential solutions to accurate protein quantitation.

We also want to discuss the possible biological reasons for discordant mRNA and protein levels aside from IHC technical limitations (e.g., antibody binding properties, interobserver variability in interpretation, narrow range of staining intensity). Other studies have stated that discordant correlation between mRNA and protein levels can be due to regulatory elements that play diverse roles in translation [4–6]. Previous studies have also reported that several mRNA elements affect translation and mRNA stability, such as codon usage, start codon context, and among others, secondary structures [4]. Transcript range is also in four orders of magnitude, compared to eight in proteins, which explains the higher coverage of RNA-seq in comparison to mass spectrometry. In addition, the number of protein molecules produced per mRNA molecule is much higher for abundant transcripts. It is postulated that genes encoding for abundant proteins have higher mRNA levels and also encode regulatory elements that lead to high translation efficiency and protein stability. On the other hand, small proteins are difficult to measure and mass spectrometry sample preparation may also affect protein concentration [4].

The Human Protein Atlas, with more than 10 million high-resolution IHC images in its portal, is a powerful tool for scientists engaging in protein and transcript expression studies [15]. However, immunohistochemistry, while powerful in its ability for in situ protein detection at the single cell level, has its limitations which can greatly affect biological interpretation of scientific results. Our study shows how there is a discordance between the mRNA and protein abundance in the kidney dataset of the Human Protein Atlas. We then validate the Human Protein Atlas IHC data with external mass spectrometry-based proteomics datasets and demonstrate that more than 500 proteins undetected by IHC are measured by mass spectrometry. Thus, we recommend scientists to exercise caution in treating the Human Protein Atlas’ IHC data as the ‘ground truth’ for protein expression as it could have negative repercussions in research result interpretation.

## Supplementary Information

Below is the link to the electronic supplementary material.

Supplementary file1 (XLSX 44 KB)

## Data Availability

All data analyzed in this study are publicly available and cited within the manuscript.
